# Solid Ectopic Cervical Thymus: A Case Report

**DOI:** 10.7759/cureus.25142

**Published:** 2022-05-19

**Authors:** Ashraf A Alnosair, Lojain A Alnosair, Ali A Almohammed Saleh, Ali R Al Zaid, Alanoud S Al Alhareth, Fatimah S Alkhars

**Affiliations:** 1 Pediatric Surgery, Almoosa Specialist Hospital, Al-Ahsa, SAU; 2 Medical School, Imam Abdulrahman Bin Faisal University, Dammam, SAU; 3 Medical School, King Faisal University, Al-Ahsa, SAU

**Keywords:** pediatrics, neck mass, ectopic cervical thymus, ectopic thymus, thymus gland

## Abstract

The thymus gland is a lymphoid organ normally located in the superior anterior mediastinum. It can rarely present abnormally in other sites along the thymopharyngeal canal and it might cause difficulties in breathing and/or feeding. We present a case report of an ectopic cervical thymus of a 10-month-old male infant who was presented to the hospital with a swelling on the left side of his neck for nine months. Investigations raised suspicion about four differential diagnoses and a total surgical excision for histopathological confirmation was deemed mandatory. It is of great importance to consider ectopic cervical thymus in the differential diagnosis of pediatric neck masses to avoid unnecessary procedures and prevent possible complications.

## Introduction

The thymus is a lymphoid organ, located in the superior anterior mediastinum, and is responsible for the maturation and differentiation of T lymphocytes. In neonates and infants, it is larger, more active, and in a state of continuous growth until it reaches its maximum weight (30-40 g) by the age of two to three years. As children grow into adolescence, the thymus begins to involute until it becomes an atrophic gland, mostly replaced by fatty tissue [1].

Ectopic thymus is a rare, typically benign pediatric condition in which the thymic tissue is located at an unusual site along its migration route, known as the thymopharyngeal canal [2]. It occurs most commonly in the submandibular triangle, or in the lateral neck, which is called ectopic cervical thymus (ECT) [3]. In the majority of cases, ectopic thymus presents as a cystic mass, while in fewer cases, it presents as a solid mass [2].

Patients with ectopic thymus are usually asymptomatic; however, compressive symptoms may occur in some cases, presenting with feeding difficulties, breathing difficulties, or Horner’s syndrome in rare cases [4,5]. Although ECT is an uncommon condition, it should be suspected in children presenting with a unilateral neck mass to avoid unnecessary invasive interventions [6,7].

In this article, we draw our attention to the diagnosis and management of this rare condition by reporting a case of an ectopic cervical thymus confirmed postoperatively by histopathological examination in a 10-month-old male infant who presented with left neck swelling.

## Case presentation

A 10-month-old boy presented to the hospital with a swelling over the left side of the neck for over nine months. The swelling was insidious in onset with no change in size. Based on the history of the presenting illness, he had no history of fever, snoring, or any difficulties in feeding or breathing. Family history and past medical history of the patient were insignificant with no history of radiation exposure. He was a full-term neonate with no peripartum complications. 

Upon physical examination, the patient was vitally stable with normal height and weight. Examination of the neck revealed a left cervical swelling, adjacent to the left sternocleidomastoid muscle measuring approximately 2-3 cm. The swelling was freely mobile, non-tender, non-pulsatile, and non-compressible, with no skin changes. 

**Figure 1:**
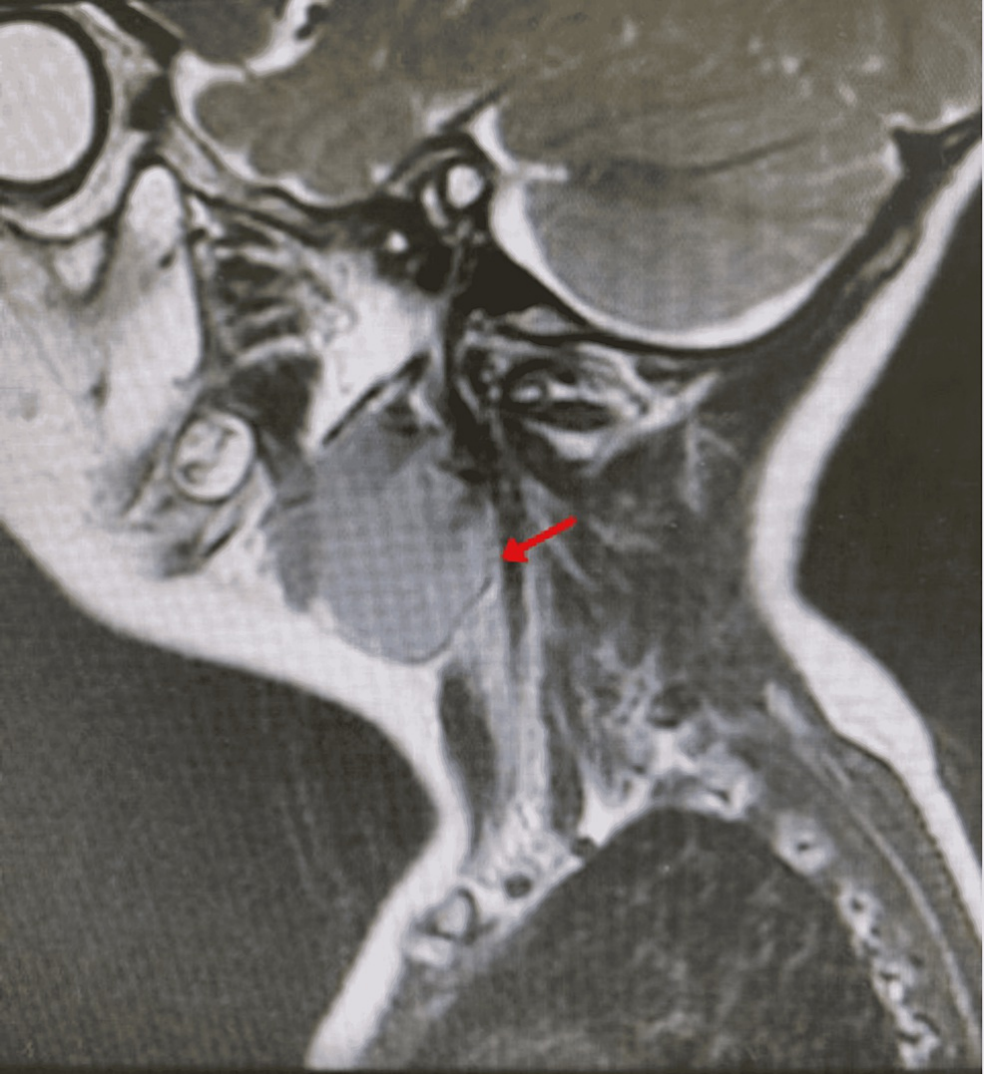
T2 head and neck MRI in sagittal view showing isointense mass in the submandibular region suggesting ectopic thymus (red arrow).

An ultrasound of the neck was performed illustrating a well-defined soft tissue mass in the left upper aspect of the neck, medial to the sternocleidomastoid muscle, and anterior to the carotid bifurcation. It measured 24x15.5mm, with internal vascularity. Further assessment was indicated; thus, an MRI was carried out. It revealed a well-circumscribed lesion on the left side of the neck measuring 3.5 x 2.3 x 2.2 cm in largest diameters. The lesion was located anterior to the sternocleidomastoid muscle, pushing the left submandibular gland anteriorly. It was superficial to the carotid sheath space, slightly pushing it posterio-laterally (Figure 1). The differential diagnoses of the swelling included neuroblastoma, lymphoma, teratoma, and an ectopic thymus. Therefore, total excision of the mass was indicated for histopathological confirmation. 

**Figure 2:**
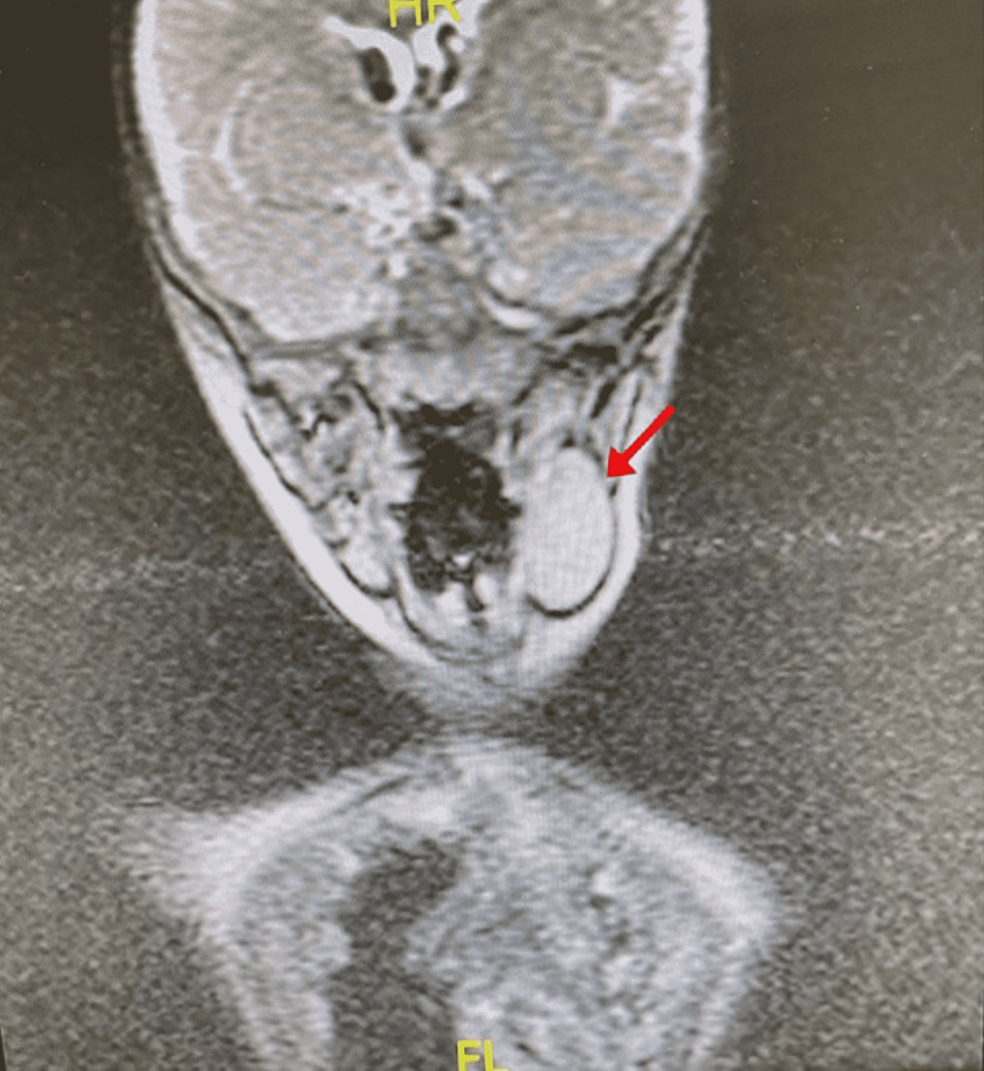
T2 head and neck MRI in coronal view showing isotense mass in the submandibular region suggesting ectopic thymus (red arrow).

Under general anesthesia, total excision of the mass was performed successfully with no perioperative complications, and the excised lesion was sent for histopathological examination. 

Upon gross examination, the soft tissue mass was solid in nature, oval-shaped, nodular, well-circumscribed, pink to dusky-blue in color, with increased vascularization. Microscopic examination reported that the excised lesion was in fact a thymic tissue with normal architecture and variably sized lobes and lobules. Some of the lobules appeared to have a thinner cortex and were associated with tingible body macrophages indicating mild involution. Small Hassall’s corpuscles were seen in the medulla with calcification and little necrotic debris. The final diagnosis of this case was confirmed to be a solid ectopic cervical thymus. 

 The patient was doing well and was discharged on day two post-operatively. On follow-up visits, the child was in a good state of health with no apparent complications, recurrence, or any complaints.

## Discussion

The thymus is a paired organ that develops from the ventral saccules of the third and occasionally, the fourth pharyngeal pouches during the sixth week of embryonic life. The primordial cells start to move medially and caudally to the thyroid in the eighth week of fetal life. The bilateral thymic primordia descend behind the sternum into the superior mediastinum after they fuse in the midline [8]. It gains its largest size at the age of three years and it keeps growing to attain its largest weight by early puberty. The thymus then begins to involute gradually during adulthood [9,10]. In infants and children, the thymus plays a significant role in the immunological mechanisms of the body and even in the prevention of autoimmune diseases [11]. Ectopic thymus is considered a rare clinical finding. It can occur in the neck, lung, pleura, or in other different locations in the thoracic cavity. It commonly occurs in the cervical region of the neck; hence, it's called ectopic cervical thymus (ECT) [12]. An aberrant ectopic cervical thymus is a rare finding and always manifests between childhood and early adulthood [9]. As the primordial thymus descends into the mediastinum during embryogenesis, a remnant of thymic tissue can be implanted along the cervical pathway of the thymopharyngeal duct, which presents as a cervical mass [13]. Although ECT can macroscopically present as either cystic or solid forms, the cystic form is more frequently reported and accounts for 76-92% of ECTs [9,14-16]. Originally, Speer described five different mechanisms that aid the process of cyst formation such as remnants of embryological thymopharyngeal ducts or degeneration of Hassall’s corpuscles [17]. In our case, the mass was solid which is rarely reported in the literature and represents only 10% of ectopic thymic masses [18]. The pathogenesis of solid ECTs involves the failure of the thymus gland to descend or its sequestration and failure to involute [19]. 

Clinically, ECT in children presents as a firm, asymptomatic neck mass with no specific clinical features. Only 6% of patients could have symptoms due to compression of the trachea which may result in stridor and dyspnea and/or compression of the esophagus which may lead to dysphagia [20,21]. It's unusual to diagnose ECT below the age of two years since it commonly occurs between 2-13 years old [16]. Our patient was a 10-month-old which is an uncommon age to be diagnosed with ECT. The differential diagnosis of a cervical mass in children includes congenital or acquired neck lumps such as thyroglossal duct cysts, dermoid and sebaceous cysts, cervical lymphadenopathy, and benign tumors such as hemangioma [22]. 

Ultrasound is considered the first-line imaging modality in diagnosing cervical ectopic thymus, as ECT is characterized by the presence of echogenic flecks in a hypoechoic background known as the “Starry Sky Appearance” [4]. Computed tomography (CT) scans and magnetic resonance imaging (MRI) are frequently needed for further assessment and anatomical clarification of the mass. MRI may detect a connection extending from the cervical thymus to the mediastinal thymus, aiding in the diagnosis of ECT [4,22,23]. The density of a solid ectopic thymus appears identical to that of a mediastinal thymus on an MRI; however, it is also similar to other lymphatic structures. Thus, it is difficult to diagnose an ectopic thymus preoperatively even on MRI [4,24].

The sole confirmatory method for the diagnosis of ectopic cervical thymus is via histopathological examination [4]. The presence of Hassall’s corpuscles on microscopic examination, as found in our case, is a pathognomonic feature for an ectopic thymus. Other characteristics may include inflammatory infiltration, cholesterol granulomas, and lymphoid follicles [21,25]. Managing ECT conservatively carries a risk of developing into malignancy [26]. Hence, thymectomy is the treatment of choice for managing cervical ectopic thymus as it is both diagnostic and therapeutic [22,26]. However, thymectomy during childhood might impact the cell-mediated immunity later on in life leading to a subsequent immunodeficiency. Thus, the presence of a mediastinal thymus should be confirmed prior to the removal of an ectopic thymus to prevent such complications [18,22,23].

## Conclusions

Ectopic cervical thymus is a rarely diagnosed condition that should be considered in the differential diagnosis of pediatric unilateral neck masses to prevent unnecessary diagnostic and therapeutic procedures. Ultrasonography should be the first modality of diagnosis followed by MRI. In difficult cases, fine needle aspiration cytology can be considered to confirm the diagnosis. Although it is rarely symptomatic, surgical excision is recommended to avoid compression symptoms which can lead to fatal outcomes, such as dyspnea and dysphagia. However, the presence of a normal mediastinal thymus gland should be confirmed prior to the surgery to avoid total thymectomy.
